# Diagnosing alkaptonuria-related nephropathy with urine albumin analysis

**DOI:** 10.1007/s00467-024-06653-6

**Published:** 2025-01-21

**Authors:** Aliye Gülbahçe, Ahmet Muderrisoglu

**Affiliations:** 1 Kocaeli City Hospital, Pediatric Nutrition and Metabolism Clinic, Kocaeli, Turkey; 2 Faculty of Medicine, Department of Pharmacology, Kırıkkale University, Kırıkkale, Turkey

**Keywords:** Albumin, Alkaptonuria, False positive, Nephropathy, Nitisinone, Proteinuria

## Abstract

Homogentisic acid (HGA) accumulation in alkaptonuria (AKU) causes injuries in various organs including the kidney. We present a case of a 9-year-old girl initially diagnosed with AKU-related nephropathy due to proteinuria found in her urine analyses. Despite 1 month of ramipril treatment, the patient’s proteinuria progressed, and as a result, kidney biopsy and nitisinone treatment were planned. In addition, we controlled her proteinuria with urine albumin measurement. After finding a normal urine albumin level in the 24-h urine sample, we realized that the patient was misdiagnosed with proteinuria because of HGA interference with urine protein level measurement using benzethonium. The patient’s treatment for kidney injury was canceled. Urine albumin level was measured as normal 2 months later, confirming proteinuria was a false-positive test result. AKU-related nephropathy should be evaluated with urine albumin measurement instead of protein. In this way, the complication risk of unnecessary interventions and pharmacotherapies can be avoided.

## Introduction

Alkaptonuria (AKU) is a tyrosine metabolism disorder that is caused by pathogenic variants in the *HGD* gene such as c.674 G > A (OMIM#203,500). Lack of homogentisate 1,2-dioxygenase enzyme (HGD) in AKU causes homogentisic acid (HGA) accumulation, leading to tissue damage including kidney injury. It progresses with age, and symptoms worsen due to the increasing HGA accumulation. Although AKU can be diagnosed in newborns, most AKU patients are diagnosed after some time with the manifestation of its symptoms which include dark urine, ochronosis, and early-age onset osteoarthropathy. Because blood HGA level has been shown to be associated with dietary protein intake, AKU is treated primarily with a protein-restricted diet. Ascorbic acid is also used because it mitigates tissue damage by inhibiting the conversion of homogentisic acid to benzoquinone acetic acid. Other drug selections are made according to the observed symptoms and organ involvement [1].

We present a case of a patient with an AKU-related nephropathy misdiagnosis due to a false-positive proteinuria test result.

## Case presentation

An 8-year-old female patient, whose parents are in a consanguineous marriage, applied to our clinic with the complaint of dark urine. She had no other complaints, and no other findings were observed in her physical examination. She was diagnosed with AKU after the HGA level was measured at 1654.8 mg/g/creatinine in her urine analysis. Her diagnosis was finalized when genetic analysis revealed that she was a carrier of the variant homozygous genotype for the *HGD* c.674 G > A mutation. Protein-restricted diet and a 500 mg daily dose of ascorbic acid therapy were initiated for her treatment.

During her control examination at the age of 9, she described dysuria. Because of this, a spot urine analysis was performed, and a high protein level in the urine was detected. A subsequent 24-h urine analysis confirmed the finding of proteinuria as well (20 mg/m^2^/h). The patient’s urine analysis revealed no other findings, and the oxalate/creatinine ratio was 0.028. A kidney ultrasound showed no kidney stone or crystal. In addition, no electrolyte or acid–base imbalances were present in the patient, and her urine concentrating ability was normal. Test results were consulted by a pediatric nephrologist and ramipril, an angiotensin-converting enzyme inhibitor with an antiproteinuric effect, and treatment was initiated. Despite 1 month of treatment, her proteinuria progressed (39 mg/m^2^/h). This showed that she had treatment-resistant proteinuria. Because there was no other possible reason for treatment-resistant proteinuria found in the patient’s history, we planned a kidney biopsy to determine the cause. Since the kidney biopsy is the definitive test for diagnosing tubular injury, we did not consider checking any urine tubular protein. In addition, tyrosine catabolism inhibitor nitisinone treatment was planned for the treatment-resistant proteinuria with no probable cause. On the other hand, we measured urine albumin levels to reevaluate proteinuria. After finding a normal urine albumin level (0.1 mg/m^2^/h) in the 24-h urine sample, we concluded that the patient was misdiagnosed with proteinuria. A subsequent literature search showed that HGA interference with urine protein measurement using benzethonium can cause false-positive proteinuria results [2]. Hence, we canceled our plans for kidney biopsy and nitisinone treatment. Additionally, the ramipril treatment was stopped. At the patient’s follow-up 2 months later, urine albumin level was measured as 0.08 mg/m^2^/h in the 24-h urine sample, showing there was never a development of a nephropathy.

## Discussion

Elevated renal clearance of HGA by both glomerular filtration and tubular secretion in patients with AKU increases the risk of nephrolithiasis by exposing the renal parenchyma and collecting system to high concentrations of HGA. With rising age, kidney functions progressively decrease with increasing HGA accumulation. Kidney impairment rarely develops, mostly after childhood [3].

Because most AKU patients develop kidney injury after childhood [1], we assumed that it was early for the manifestation of AKU-related nephropathy in this 9-year-old patient. Our assumption was indicated to be wrong at first when the patient’s urine analysis using benzethonium chloride assay showed proteinuria. By measuring urine albumin level, we realized that the first tests showed false-positive proteinuria. Sykes et al. reported that HGA causes interference in the benzethonium chloride assay for urinary protein by forming into oxidized HGA in the final alkaline reaction mixture [2]. Sykes et al. also noticed that at a fixed albumin concentration, measured urinary protein increases as HGA increases in the same measurement method [2]. These findings indicate that diagnosis of nephropathy should be made with the measurement of urine albumin, not with urine protein in AKU patients who are known to have increased urine HGA. Indeed, HGA interference caused a false-positive proteinuria result leading to a diagnosis of the patient with nephropathy, which was later realized to be a misdiagnosis with the measurement of urine albumin in this case.

Surveillance of AKU is mostly performed by physical examination and evaluation of organ involvement [1]. Thus, biochemical test results can sometimes be overlooked. Further, misleading test results can hinder AKU management. False-positive result of proteinuria caused unnecessary use of ramipril for a month and almost unnecessary use of nitisinone in this case. Although both drugs are known to be safe, ramipril can cause fainting in 10% of patients due to hypotension, and nitisinone can rarely cause some serious side effects such as thrombocytopenia and leucopenia [4]. Therefore, these drugs should only be used when necessary. By reevaluating proteinuria with urine albumin measurement, the unnecessary uses of ramipril and nitisinone were avoided in this case. In addition, we canceled our kidney biopsy plan which is an invasive procedure with complications such as bleeding and infection.

In conclusion, AKU-related nephropathy should be evaluated with urine albumin measurement rather than measuring urine protein. In this way, complications of unnecessary interventions and pharmacotherapies can be avoided in AKU management.

## Summary

### What is new?

- Evaluating AKU-related nephropathy with urine protein analysis can lead to misdiagnosis. To avoid unnecessary invasive procedures and treatments, AKU-related nephropathy should be evaluated with urine albumin analysis.

## Data Availability

All data supporting the findings of this study are available within the paper.

## References

[1] Bernardini G, Braconi D, Zatkova A, Sireau N, Kujawa MJ, Introne WJ, Spiga O, Geminiani M, Gallagher JA, Ranganath LR, Santucci A (2024) Alkaptonuria. Nat Rev Dis Primers 10:16. 10.1038/s41572-024-00498-x 38453957 10.1038/s41572-024-00498-x

[2] Sykes E, Gibson M, Dmuchowski C (1996) Homogentisic acid interference in the measurement of urinary protein using benzethonium chloride. Ann Clin Biochem 33:86–88. 10.1177/000456329603300117 8929077 10.1177/000456329603300117

[3] Mullan A, Cocker D, Taylor G, Millar C, Ranganath L (2015) Fatal oxidative haemolysis and methaemoglobinaemia in a patient with alkaptonuria and acute kidney injury. Clin Kidney J 8:109–112. 10.1093/ckj/sfu121 25713720 10.1093/ckj/sfu121 PMC4310424

[4] Datapharm Ltd (n.d.) Electronic medicines compendium. https://www.medicines.org.uk/. Accessed 14 Dec 2024

